# The N-Terminal Fragment of a PB2 Subunit from the Influenza A Virus (A/Hong Kong/156/1997 H5N1) Effectively Inhibits RNP Activity and Viral Replication

**DOI:** 10.1371/journal.pone.0114502

**Published:** 2014-12-02

**Authors:** Takahito Kashiwagi, Koyu Hara, Yoko Nakazono, Yusaku Uemura, Yoshihiro Imamura, Nobuyuki Hamada, Hiroshi Watanabe

**Affiliations:** Department of Infection Control and Prevention, Kurume University School of Medicine, Fukuoka, Japan; Rutgers, The State University of New Jersey, United States of America

## Abstract

**Background:**

Influenza A virus has a RNA-dependent RNA polymerase (RdRp) that is composed of three subunits (PB1, PB2 and PA subunit), which assemble with nucleoproteins (NP) and a viral RNA (vRNA) to form a RNP complex in the host nucleus. Recently, we demonstrated that the combination of influenza ribonucleoprotein (RNP) components is important for both its assembly and activity. Therefore, we questioned whether the inhibition of the RNP combination via an incompatible component in the RNP complex could become a methodology for an anti-influenza drug.

**Methodology/Principal Findings:**

We found that a H5N1 PB2 subunit efficiently inhibits H1N1 RNP assembly and activity. Moreover, we determined the domains and important amino acids on the N-terminus of the PB2 subunit that are required for a strong inhibitory effect. The NP binding site of the PB2 subunit is important for the inhibition of RNP activity by another strain. A plaque assay also confirmed that a fragment of the PB2 subunit could inhibit viral replication.

**Conclusions/Significance:**

Our results suggest that the N-terminal fragment of a PB2 subunit becomes an inhibitor that targets influenza RNP activity that is different from that targeted by current drugs such as M2 and NA inhibitors.

## Introduction

The influenza A virus belongs to the family *Orthomyxoviridae* and has eight segmented RNA-genomes, which can cause a genetic reassortment that can generate new pandemic influenza A viruses such as H1N1, H2N2, and H3N2 subtypes [1]. Generally, it is believed that a new influenza A virus emerges from swine with co-infections of more than two different influenza A viruses [2]. Mathematically, 256 ( = 2^8^) types of new influenza viruses can be generated when eight genomes derived from two different influenza A viruses are merged into one viral particle in a host animal. On the other hand, recent studies have shown that a genetic reassortment of the influenza A virus is restricted by an incompatible combination of ribonucleoprotein complex (RNP) in animal cells [3]–[7]. In fact, over the past century, only 4 strains have been allowed to emerge as pandemics [1].

Influenza A virus has a RNA-dependent RNA polymerase (RdRp) constituted from three subunits (PB1, PB2 and PA subunit), which assembles with nucleoproteins (NP) and a viral RNA (vRNA), forming a RNP complex in the host nucleus [1], [8]. Recently, our research has shown that an incompatible combination of RNP components, e.g., A/WSN/33 (H1N1) (WSN as abbreviation) PB1, WSN PA and A/HongKong/156/97 (H5N1) (HK as abbreviation) PB2 subunit, severely lost the RNP activity in a HEK 293T cell [4], which indicates that a combination of the RNP components is important for RNP assembly and activity. In a similar manner, other groups have suggested a potential role for the combination of RNP components for RNP activity [5], [6]. Moreover, some groups have reported that the short peptides that disrupt the assembly of a polymerase have shown an inhibitory effect on RNP activity [9]–[11]. These reports indicate that the inhibitor for influenza RNP assembly can also become the target for a new class of anti-influenza drugs that could take the place of neuraminidase (NA) inhibitors.

Influenza A virus is surrounded by two surface glycoproteins hemagglutinin (HA) and neuraminidase (NA). As a therapy for influenza, NA inhibitors were a dramatic development [12]–[18]. In Japan, four kinds of neuraminidase inhibitors are presently acceptable for therapy, although these drugs show the same active mechanism, which raises concerns of drug resistance. In fact, Russian H1N1, which was previously a seasonal strain, developed resistance to these drugs, and was easily spread throughout the world [19],[20]. Surprisingly, only one amino acid substitution in the NA was needed to obtain resistance [21], [22]. Therefore, a new drug with a mechanism that is unlike that of NA inhibitors is strongly desired in the world. Recently, a new drug to treat influenza RNA polymerase, Favipiravir (T-705), has been developed, and is expected to be a popular new choice for anti-influenza therapy [23], [24].

The results of a previous study have shown that the avian H5N1 influenza PB2 subunit severely impairs human H1N1 RNP assembly and activity [4]. Therefore, in the present study we applied the H5N1 PB2 subunit as a inhibitor against influenza RNA polymerase. We demonstrated that a H5N1 PB2 subunit could effectively inhibit not only H1N1 but also H5N1 RNP activity. Moreover, we determined the domains and important amino acids on the N-terminus of the PB2 subunit that are required for an effective inhibitory effect. Plaque assay also confirmed that the N-terminal fragment of a H5N1 PB2 subunit could inhibit viral replication. Our data suggest that the N-terminal fragment of a H5N1 PB2 subunit could be a good agent for new therapies against the influenza A virus, because of the different mechanisms of the drug that are based on its NA inhibitors.

## Materials and Methods

### Strains and plasmids

cDNA clones isolated from the following influenza strains were used in this report: A/WSN/33 (H1N1) (abbreviated as WSN) and A/HongKong/156/97 (H5N1) (abbreviated as HK) [25], [26].

PB1, PB2, PA and NP expression plasmids of influenza viruses WSN and HK have previously been described [25]–[27]. Briefly, each of the coding regions were reverse-transcribed from the isolated RNA, and were amplified by specific primers (RT-PCR). PCR fragments were digested by the proper restriction enzymes and inserted into the multi-cloning site of the pcDNA(+) 3.1 vector (Invitrogen), generating pcDNA/WSN/PB1, pcDNA/HK/PB1, pcDNA/WSN/PB2, pcDNA/HK/PB2, pcDNA/WSN/PA, pcDNA/HK/PA, and pcDNA/WSN/NP. The pPOLI/WSN/vNA that expresses a viral RNA of the influenza NA gene has also been described [28].

In order to construct a deleted- or point-mutated PB2 subunit, site-directed mutagenesis described in the previous report [25], [29] was performed. Either the point-mutated primer pairs for the point mutation or the primer pairs including the end-sequences of binding regions for the deleted mutant were used. The amplified PCR products with a vector sequence were transfected and circularized in the competent cell (DH5-alpha, TOYOBO, Japan), generating the expression vectors containing the point or deleted mutants. To confirm these sequences, the coding regions were fully sequenced by the outside order (FASMAQ co., Japan).

### Luciferase reporter and dual luciferase reporter assays

To screen the RNP activity by luciferase activity, a pPOLI/vLUC vector was constructed by substituting the influenza NA gene in the pPOLI/WSN/vNA vector with the luciferase gene of a firefly. The subconfluent monolayers of an embryonic kidney cell (HEK 293T) [26], [29], [30] in E-MEM medium with 10% fetal bovine serum in a 6-well plate were transfected with 0.2 µg/well each of the PA, PB1, PB2, NP, and vLUC (viral-like luciferase gene) expression vectors from each strain (WSN or HK) using Lipofectamine 2000 (Invitrogen). For the inhibition assay, 1.0 µg/well of a PB2 subunit or PB2 fragment expression vector was also transfected with the other components of influenza RNP. Cells were lysed at 30 hours after transfection either with a Cell Culture Lysis Buffer (CCLB) (Promega) for a luciferase assay or with a Passive Lysis Buffer (PLB) (Promega) for a dual luciferase assay. A reagent of luciferin (Promega) was then added to the cell lysate according to the manufacturer’s protocol. Luciferase activity was measured using a luminometer Lumat LB 9507 (Berthold, Germany) and was calculated as a relative light unit (RLU).

A dual luciferase assay was also performed to quantify the influenza RNP activity and inhibitory effect. Briefly, 0.2 µg/well of pRL-TK vector (Promega) expressing *Renilla* luciferase as an internal control was co-transfected with 0.2 µg/well each of vectors of influenza RNP components in HEK 293T cells, and the *firefly* luciferase activity was normalized by *Renilla* luciferase activity.

### RNA isolation and primer extension assay

To reconstitute the ribonucleoprotein (RNP) of the influenza A virus and analyze the RNP activity, subconfluent monolayers of 293T cells in E-MEM medium with 10% fetal bovine serum (FBS) in a 6-well plate were transfected with 0.2 µg/well each of PA, PB1, PB2, NP, and vNA (viral NA gene) expression vectors from each strain (WSN or HK) via Lipofectamine 2000 (Invitrogen). For the inhibition assay, 1.0 µg/well each inhibitor such as the fragment of a PB2 subunit expression vector was also transfected with the components of RNP. Cells were harvested at 30 hours after transfection, and total RNA was extracted using TRIzol reagent (Invitrogen). The RNA was then analyzed by a primer extension assay using three primers: one for vRNA, one for mRNA and cRNA, and one for 5S ribosomal RNA that was used as an internal control [26], [29], [30]. These transcripts were visualized by 6% polyacrylamide gel containing 7M urea in TBE buffer, and were detected by autoradiography. Each activity was standardized using 5S ribosomal RNA.

### Preparation of TAP-tagged PB2 fragment and TAP purification

To confirm the binding ability of a PB2 fragment to a PB1-PA dimer and to a RNP, a TAP-tagged PB2 fragment was prepared. For preparation of the polymerase, 293T cells were harvested in 6-well plates, and transfected with 0.2 µg/well each of expression vectors containing WSN/PA, WSN/PB1, and TAP-tagged HK/PB2 fragment by using Lipofectamine 2000 (Invitrogen). For the preparation of the RNP complex, WSN/NP, and WSN/vNA expression vectors were also transfected simultaneously. Cells were collected at 40 hours post-transfection and the proteins were extracted and purified using the tandem affinity purification (TAP) method that was described previously [27]. Partially purified proteins were analyzed by 12% SDS-PAGE using silver staining (Invitrogen) and were confirmed by western blotting with specific anti-bodies against PB1, PB2, PA, NP, and TAP [31], [32].

### Plaque assay

To assess the inhibitory effect of a HK/PB2 fragment on viral replication, an HK/PB2 fragment was expressed in advance in a MDCK cell. Briefly, MDCK cells were transfected with either 0–2.0 µg/well expression vectors of a HK/PB2 fragment or a WSN/PB2 subunit and were incubated at 37°C with 5% CO_2_ for 24 h in a 6-well plate. After the incubation, a plaque assay was performed using 50 PFU/well of a WSN strain. The plaque-forming number was counted for each well, and the relative inhibitory effects of the HK/PB2 fragment and the WSN/PB2 subunit were calculated.

## Results

### Inhibitory effect of a HK (H5N1) PB2 subunit against WSN (H1N1) RNP

**Figure 1:**
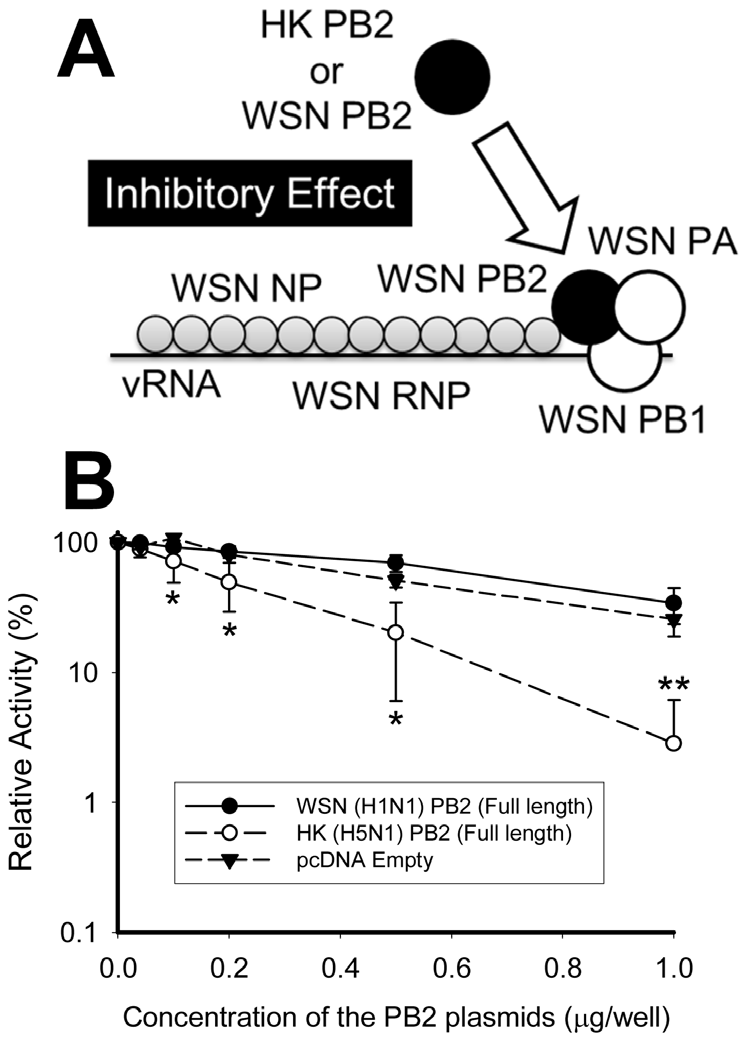
Inhibitory effects of WSN and HK/PB2 subunits against WSN/RNP activity. (A) The model of the study for the inhibitory effect by the PB2 subunit against WSN/RNP activity. (B) Inhibitory effects of HK (H5N1) and WSN (H1N1) PB2 subunits. Closed and opened circles indicate WSN (H1N1) and HK (H5N1) PB2 subunits, respectively, expressed as inhibitors. The triangle indicates pcDNA/empty vector as a negative control. WSN RNP was reconstituted with either HK (H5N1) or WSN (H1N1) PB2 subunits in 293T cells using a constant concentration of the vectors of RNP components (0.2 µg/well each of pcDNA/WSN/PB1, pcDNA/WSN/PB2, pcDNA/WSN/PA, pcDNA/WSN/NP, and pPOLI/vLUC) and varied concentrations of the vectors of inhibitors (from 0.04 to 1.0 µg/well of either pcDNA/HK/PB2, pcDNA/WSN/PB2, or pcDNA/empty). The inhibitory effects of them were estimated by luciferase reporter assay. * and ** indicate statistically significant differences at <0.05 and <0.01, respectively, in a Student’s t-test (n = 3).

In a previous report we showed that an incompatible combination of RNP components, e.g., A/WSN/33 (H1N1) (WSN as abbreviation) PB1, WSN/PA and A/HongKong/156/97 (H5N1) (HK as abbreviation) PB2 subunit, severely lost the RNP activity in a HEK 293T cell [4]. Therefore, we first confirmed whether this HK/PB2 subunit could inhibit WSN/RNP (Figure 1A) activity. When WSN/RNP was co-expressed with HK/PB2 in a 293T cell, WSN/RNP activity was dose-dependently inhibited by the expression vector of the HK/PB2 subunit. Though WSN/PB2 also showed a small reduction, the non-specific reduction was the same level as that of the empty vector (Figure 1B). Significant reductions in RNP activity were observed when 0.1–1.0 µg/well of the expression vector of HK/PB2 were co-transfected as an inhibitor in a HEK 293T cell compared with those of WSN/PB2 and empty vectors. Thus, we could confirm that the HK/PB2 subunit effectively inhibits WSN/RNP activity, and that the inhibitory effect is significantly strong.

### Screening of the inhibitory effect of the fragments derived from a HK/PB2 subunit

**Figure 2:**
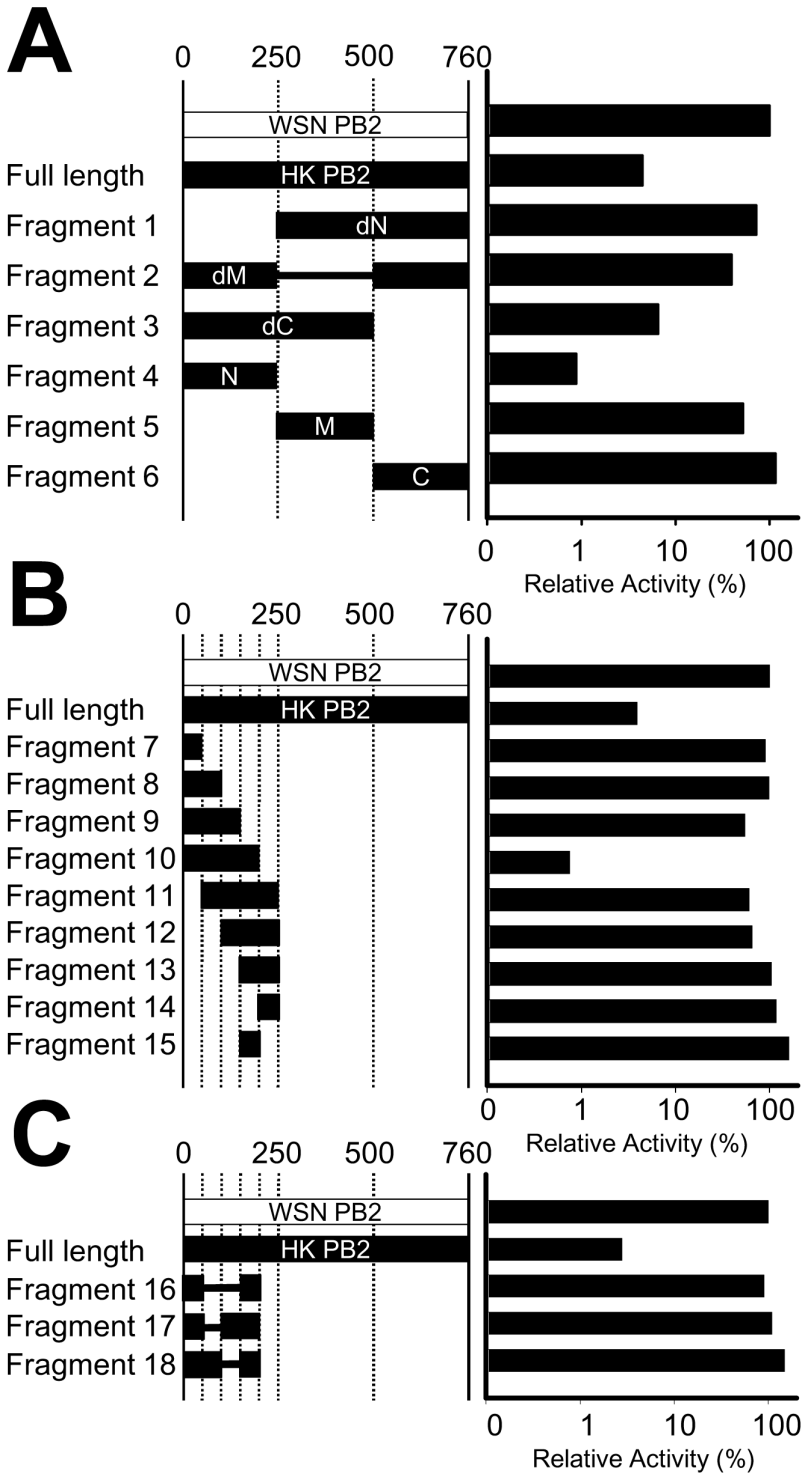
Screening of the inhibitory effect using inhibitors derived from a HK/PB2 subunit. (A) Deleted mutants of the C, middle and N-terminus of the HK/PB2 subunit. (B) Truncated mutants from fragment 4 in panel A. (C) Deleted mutants from fragment 10 in panel B. Fragment numbers and regions used in this test are indicated by the left side of the panel. The WSN/RNP activities with each inhibitor were measured by a luciferase reporter assay, and the averages were calculated from two independent trials. The WSN PB2 subunit, shown as a white bar, was used as a negative control. The designations dN, dM and dC indicate the deletion of the N-terminus (1 to 250 amino acids), Middle (251 to 500 amino acids) and C-terminus (501 to 760 amino acids) of the HK/PB2 subunit, respectively. N, M and C contain only the N-, middle- and C-terminus of the HK PB2 subunit, respectively.

To screen which region of the HK/PB2 subunit is required for the effective inhibition of WSN/RNP activity, we constructed the various fragments of a HK/PB2 subunit (Figure 2A), and the WSN/RNP activities were measured with each fragment as inhibitors. The RNP activity was strongly inhibited by fragments 3 (dC) and 4 (N). On the other hand, fragments 1 (dN), 5 (M) and 6 (C) only showed either a small or no inhibitory effect. These results indicate that the N-terminus of a PB2 subunit is important for a reduction in RNP activity. Though fragment 2 (dM) contained an N-terminal PB2 subunit, its RNP activity was not inhibited, as with fragments 3 and 4.

To further confirm which region of an N-terminal (from 1 to 250 amino acids) HK/PB2 subunit is required in order to reduce the WSN/RNP activity, a truncation at every 50 amino acids on the N-terminal HK/PB2 subunit allowed a co-transfection with WSN/RNP into a 293T cell (Figure 2B). The WSN/RNP activity was strongly inhibited by only fragment 10, which contained 1–200 amino acids of an N-terminal HK/PB2 subunit, although both fragments 9 (1–150) and 11 (50–250) showed only a small reduction. The WSN/RNP activities were not severely reduced by either fragments 7 (from 1 to 50) or 15 (from 150 to 200), although fragment 15 showed a small reduction.

To obtain fragments that were as small as possible, the regions between 1 to 50 and between 150 to 200 amino acids were removed from fragment 10, which generated fragments 16, 17 and 18 (Figure 2C). When these fragments were co-expressed by the WSN/RNP in the HEK 293T cells, the activities of the WSN/RNP did not decrease. Therefore, we decided to use fragment 10 (Frag.10) as an inhibitor in the next experiment.

### Confirming the inhibitory effect of Frag.10 against the other subtypes of RNP activities

**Figure 3:**
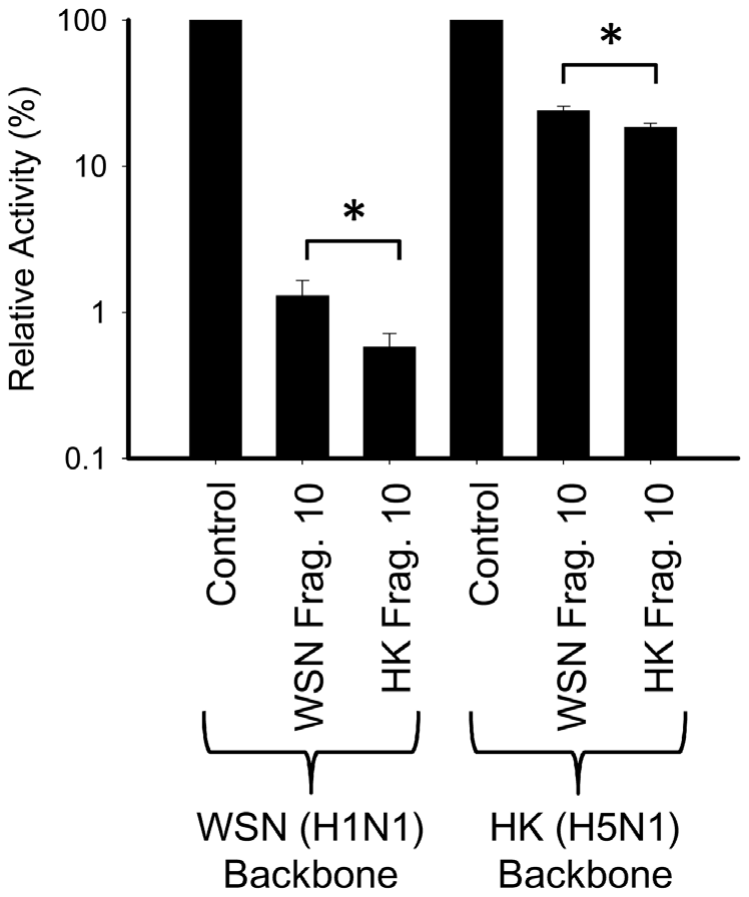
Inhibitory effect of Frag.10 against the RNP activity from another strain. The intra- and interspecific effects of WSN and HK/Frag.10 against either WSN or HK/RNP activities were tested. WSN/Frag.10 was also constructed as well as that of HK/Frag.10 used in the previous screening. The RNP activities were measured by dual luciferase reporter assay, then the *Firefly* luciferase activity was normalized by *Renilla* luciferase activity. The relative activities of WSN and HK/RNP without the inhibitory peptide are expressed as 100% activity. The standard deviations and significant differences were calculated from three independent trials. * indicates statistically significant differences at <0.05, in a Student’s t-test (n = 3).

In order to confirm whether Frag.10 can inhibit the RNP in the other subtypes, a WSN/Frag.10 that was derived from WSN/PB2 was also constructed. Expressing either WSN or HK/RNP in a 293T cell, the RNP activities were measured with WSN or HK/Frag.10 via dual-luciferase reporter assay. pRL-TK (Promega) expressing *Renilla* luciferase was used as an internal control and *firefly* luciferase activity was normalized by *Renilla* luciferase activity. As shown in Figure 3, the activity of both WSN and HK/RNP was severely impaired by WSN and HK/Frag.10. When WSN and HK/Frag.10 were compared, the inhibitory effect of HK/Frag.10 was significantly higher than that of WSN.

### Determination of the amino acid that is important for the strong inhibitory effect of HK/Frag.10

**Figure 4:**
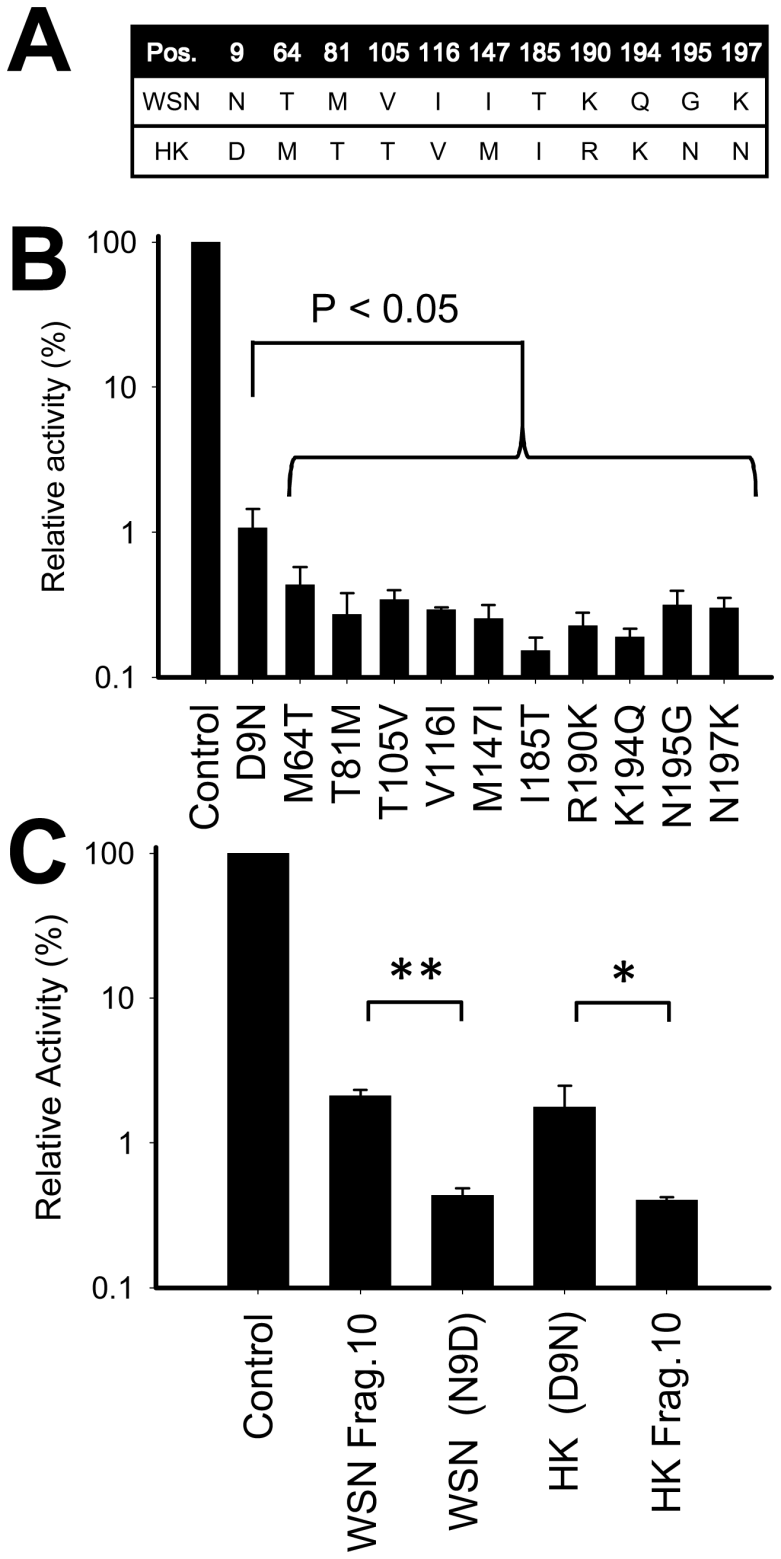
Searching important amino acid for an inhibitory effect. (A) The compared differences of the amino acids between WSN and HK/Frag.10 are shown. (B and C) The WSN/RNP activities are expressed as a % relative to the WSN/RNP activity without the inhibitors (Control). The activities were measured via luciferase (B) and dual luciferase assay (C). The standard deviations and significant differences were calculated from three independent trials. * and ** indicate statistically significant differences at <0.05 and <0.01, respectively, in a Student’s t-test (n = 3).

To determine which amino acid contributes the most to the difference, we aligned the peptides between WSN and HK/Frag.10 and found 11 amino acid changes in the fragments (Figure 4A). Using a site-directed mutagenesis method, each amino acid on HK/Frag.10 was substituted with its counterpart on WSN, and the inhibitory effects of the mutants were measured via luciferase reporter assay (Figure 4B). These substituted fragments showed strong inhibitory effects, with the exception of fragment D9N (the aspartic acid was substituted for the asparagine at position 9 on the HK/Frag.10), for which the inhibitory effect was significantly attenuated.

To further confirm the importance of the position 9 on the fragment, WSN/Frag.10 (N9D) was also constructed, and the inhibitory effects were compared with those of HK and WSN/Frags.10 (wild type) via dual luciferase assay (Figure 4C). The inhibitory effect of WSN/Frag.10 (N9D) was significantly increased by the point mutation of the position 9, while that of HK/Frag.10 (D9N) was complementarily attenuated.

### Confirming the protein expression levels of WSN/RNP components, and binding ability of the HK/Frag.10 to the other subunits (WSN/PB1, WSN/PA and WSN/NP)

**Figure 5:**
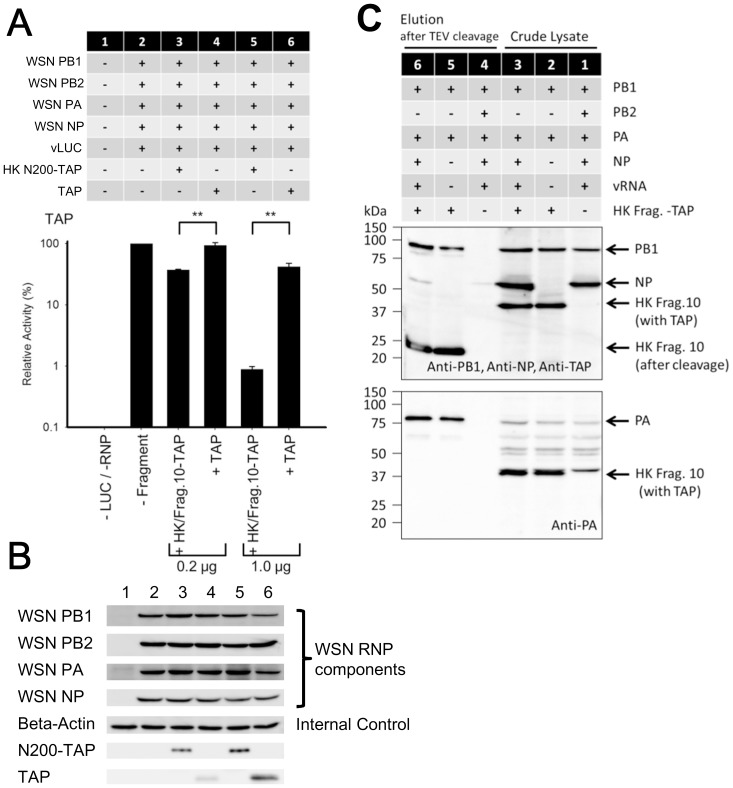
Confirmation of the binding to other subunits. (A) The inhibitory effect of HK/Frag.10-TAP was confirmed. The RNP activities are expressed as a % relative to the WSN/RNP activity without the inhibitor. The standard deviations were calculated from three independent trials. ** indicates statistically significant differences at <0.01, using a Student’s t-test (n = 3). (B) Protein expression levels of WSN/RNP components and HK/Frag.10-TAP were confirmed by western-blotting using specific antibodies. (C) Binding to the other subunit such as WSN/PB1, WSN/PB2 and WSN/NP was confirmed by co-purification and western blotting. Crude lysates (Lanes 1 to 3) were purified via the TAP-purification method (Lanes 4 to 6). The WSN/RNP without HK/Frag.10-TAP were expressed and analyzed as a negative control (Lanes 1 and 4). As trimeric components, WSN/PB1, WSN/PA and HK/Frag.10-TAP were co-expressed (Lane 2) and purified via TAP purification (Lane 5). As RNP components, WSN/PB1, WSN/PA, WSN/NP, WSN/vNA RNA and HK/Frag.10-TAP were co-expressed (Lane 3) and purified by TAP purification (Lane 6). In lanes 4 to 6, the molecular size of HK/Frag.10 was shifted from 44 kDa to 23 kDa as a result of the cleavage by TEV protease for eluting proteins from the IgG sephalose column. The table indicates which subunits and HK/Frag.10-TAP were co-transfected in 293T cell. A reproduction of the result was confirmed by two independent trials.

We first confirmed the expression levels of influenza RNP components (including WSN/PB1, WSN/PB2, WSN/PA, and WSN/NP) and that of HK/Frag. 10 in a HEK 293T cell via western blotting using specific antibodies. To detect the expression level of the fragment, we conjugated a tandem affinity purification tag to the C-terminus of HK/Frag.10 (HK/Frag.10-TAP). As a control, pcDNA/TAP was additionally constructed containing only a TAP coding region, then inhibitory effects (Figure 5A) and protein expression levels (Figure 5B) were respectively checked. In the presence of HK/Frag.10-TAP, significant inhibitions of WSN/RNP activities were observed according to the expression levels of the fragment (Figure 5A). As shown in Figure 5B, any protein expressions of influenza RNP components and beta-Actin were not affected by the HK/Frag.10-TAP.

To investigate whether the HK/Frag.10 binds to either WSN/PB1 and/or WSN/NP subunit(s), we next performed a TAP co-purification assay using HK/Frag.10-TAP, because the HK/Frag.10 retains the PB1 and NP binding sites. In the crude lysate (Figure 5C, lane 1), the protein expression of WSN/RNP was correctly confirmed by western blotting using each of the specific antibodies. When the WSN/RNP without HK/Frag.10-TAP was purified by TAP-purification, no bands were detected, with the exception of a very faint band for NP (Figure 5C, lane 4). On the other hand, the WSN/PB1 and WSN/PA subunits were co-purified with HK/Frag.10-TAP by TAP-purification, when the WSN/PB1 and WSN/PA subunits were co-expressed with the HK/Frag.10-TAP in the 293T cells (Figure 5C, lane 5). Though WSN/PA and WSN/PB1 were also detected when the WSN/PB1, WSN/PA, WSN/NP, and viral WSN/vNA genes, which are the components of the WSN/RNP, were co-expressed with the HK/frag.10-TAP (Figure 5B, lane 6), the intensity of the NP band was the same level as that of the background (Figure 5B, compare lanes 4 and 6). Incidentally, the HK/Frag.10-TAP was detected after cleavage by TEV-protease via the use of TAP-antibody obtained from GeneScript (Figure 5C, upper panel of lanes 5 and 6), because the recognition site of this commercial TAP-antibody is on the upstream peptides of the digestion site of TEV protease. HK/Frag.10-TAP before cleavage by TEV-protease was detected via the use of PA-antibody (Figure 5C, lower panel of lane 2 and 3), because the TAP-tag contains protein A that can nonspecifically capture any immunoglobulin G (IgG).

### Confirming the specificity of the inhibitory effect by WSN/RNP reconstitution and primer extension assay

**Figure 6:**
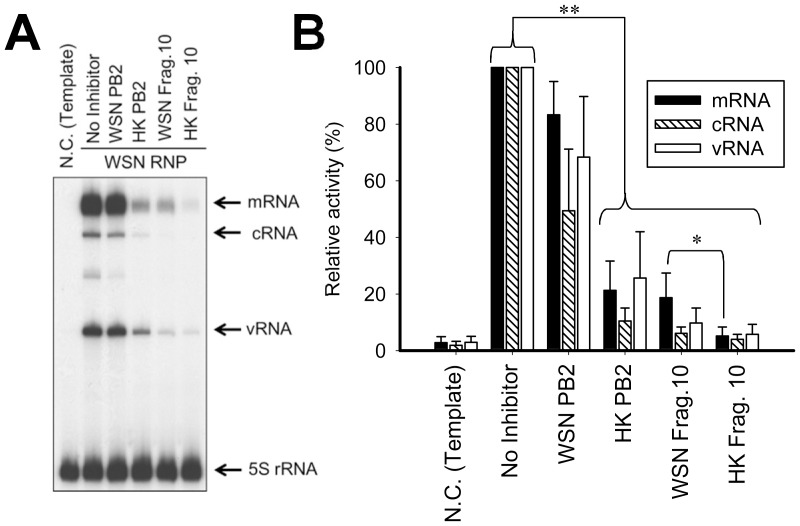
Comparison of RNP activities containing inhibitors by primer extension assay. (A) Representative analyzed polyacrylamide gel (6%) is shown. Each position of mRNA, cRNA, vRNA and 5 s rRNA as an internal control is indicated on the right. (B) Quantitation of mRNA (closed bar), cRNA (oblique-lined bar) and vRNA (opened bar) of WSN with/without inhibitors. The standard deviations were calculated from three independent trials. * and ** indicate statistically significant differences at <0.05 and <0.01, respectively, in a Student’s t-test (n = 3).

To confirm the specificity of the inhibitory effect, the WSN/RNP was reconstituted with/without inhibitors in 293T cells and the WSN/RNP activity was measured using a primer extension assay (Figure 6A and 6B). Compared with a WSN/PB2 subunit, both replication (vRNA and cRNA) and transcription (mRNA) of the WSN/RNP activity were severely impaired by a HK/PB2 subunit. As shown in a luciferase assay, a non-specific reduction was also observed by the WSN/PB2 subunit. When WSN and HK/Frag.10 were used as inhibitors, strong inhibitory effects were observed. In addition, the mRNA level of HK/Frag.10 was significantly lower than that of the WSN/Frag.10. These results agreed completely with the dual luciferase assay, as shown in Figure 3. Thus, it was confirmed that the HK/PB2 subunit and its fragment specifically decreased WSN/RNP activity.

### Using plaque assay to confirm the inhibitory effect of HK/Frag.10 against viral growth

**Figure 7:**
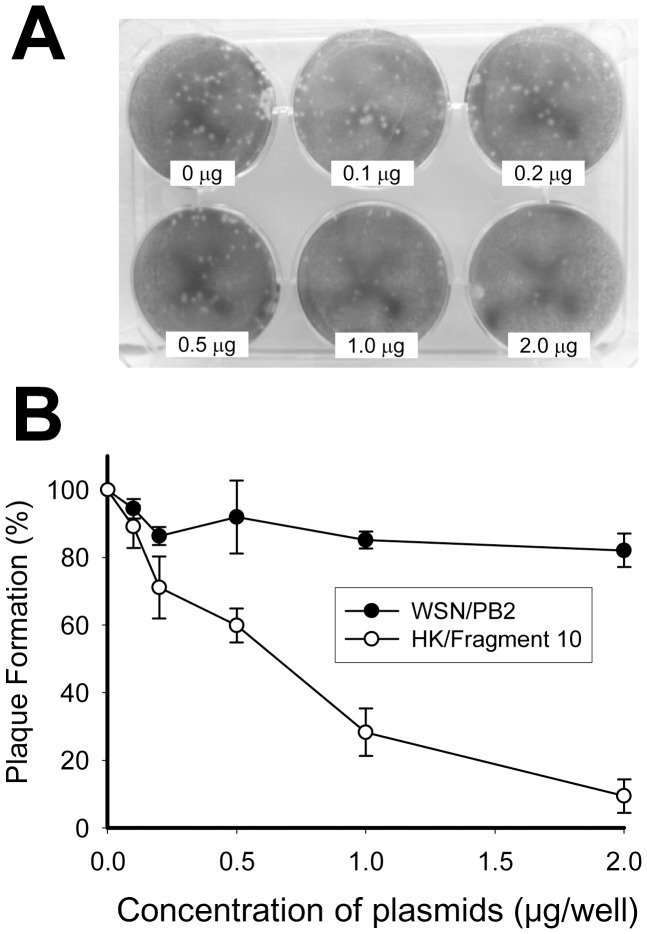
Confirmation of the inhibitory effect against viral growth by plaque assay. (A) Representative plaque assay is shown (6-well plate). (B) Quantitation of plaque forming units (PFU) is shown. Plaque formations with various concentrations of HK/Frag.10 and WSN/PB2 (from 0 to 2.0 µg/well) are expressed as a % relative to the plaque number without an inhibitor. The standard deviations were calculated from three independent trials (n = 3).

In order to confirm whether HK/Frag.10 can inhibit viral growth, a plaque assay using a WSN strain was performed (Figure 7A). Each of the concentration amounts (0 −2.0 µg/well) of plasmids expressing HK/Frag.10 or WSN/PB2 were transfected to a MDCK cell on a 6-wells plate in advance, and then 50 PFU/well of influenza virus (WSN strain) was inoculated to the MDCK cells at 24 hours post-transfection. Consequently, the viral growth was dose-dependently decreased by HK/Frag.10.

## Discussion

First, we confirmed that the HK/PB2 subunit could be used as an inhibitor to the WSN/PB2 subunit (Figure 1A). Interestingly, a strong inhibitory effect of the HK/PB2 subunit was observed (Figure 1B). It was previously shown that both polymerase activity and promoter binding to a model template were significantly promoted *in vitro*, when a HK/PB2 subunit was introduced into the dimer of WSN/PB1 and WSN/PA [4], [26], which indicated that the HK/PB2 subunit could be incorporated into the WSN/PB1-PA dimer more efficiently than that of the WSN/PB2 subunit.

Both fragments 3 and 4 contained an N-terminus of the PB2 subunit and showed a strong inhibitory effect (Figure 2A). These results indicate that the N-terminal of the PB2 subunit, which contains both PB1 and NP binding domains [33]–[36], is important to the inhibitory effect. Fragment 2 showed a small inhibitory effect, although the N-terminus of the PB2 subunit was contained. By deletion of the middle region of the PB2 subunit, the structure of fragment 2 might be materially disrupted. Though fragment 10 (HK/Frag.10) including 1 to 200 amino acids of the N-terminal PB2 subunit showed a strong inhibitory effect, the RNP activity was not inhibited by fragments 7 and 15 (Figure 2B). These results suggest that two domains, positions 1 to 50 and 150 to 200, were necessary for the inhibitory effect, but they could not individually affect the RNP activity. The WSN RNP activities were not decreased by fragments 16 to 18 (Figure 2C), although these fragments included both PB1 and NP binding domains. These results suggest that the structural distribution of both domains may also be important for the inhibition, or some distance between two domains may be needed for the effective inhibition. Recent studies have shown that host chaperonins are needed for the correct folding of viral proteins [37], [38]. These fragments from 16 to 18 may not bind to these factors because of the disruption of their primary structures.

As shown in the previous report [10], a fragment of the WSN/PB2 subunit containing only 1 to 37 amino acids inhibited WSN/RNP activity, but it did not inhibit the RNP activity derived from the other strains, because of diminished binding to the PB1 subunit. That report also suggested that the PB2 binding site on the PB1 subunit was masked by PA-PB1 binding. On the other hand, our HK/Frag.10 showed an inhibitory effect against both homo- and hetero-subtype strains (Figure 3). This result indicates that a NP binding site may be needed for the inhibitory effect against other strains; otherwise the masked PB2 binding site on a PB1 subunit might be recovered by the NP binding site of the fragment. Also, it suggests that the mechanism of inhibitory effect is more than simple competitive effect; because the fragment derived from same strain showed a strong inhibition (Figure 3). Previously, Graef *et al.* reported that PB2 subunit inhibits the expression of the host protein via the interaction of MAVS (also known as IPS-1) [39]. Thus, our fragment may indirectly affect the inhibitory effect against influenza RNP activity via the expression of host proteins in addition to direct effect. Taken together, these results suggest that HK/Frag.10 may be developed as a broad-spectrum inhibitor that can target influenza RNP activity.

Via a site-directed mutagenesis, the importance of amino acid position 9 (D9N) on the HK/Frag.10 was indicated (Figure 4). A previous report showed that this position of asparagine on the PB2 subunit contributed to the localization to mitochondria in the cell, which was not found with aspartic acid [39]. Presumably, the localization of the fragment in the cell might be changed by this substitution, thereby affecting the inhibitory effect, although we could not directly confirm this because of the lack of a detectable antibody for an untagged short fragment of the PB2 subunit.

When WSN/PB1 and PA subunits were co-expressed with HK/Frag.10-TAP, both subunits were co-purified with HK/Frag.10-TAP by TAP purification (Figure 5C lane 5). This result indicated that the HK/Frag.10 retained the ability to bind to the WSN/PB1-PA dimer [27], and could form a defective 3P complex including WSN/PB1, WSN/PA and HK/Frag.10. However, this defective 3P complex could not form a RNP complex, because the accumulation of NP was not observed, although the background level of NP was detected (Figure 5C lane 6). These results are supported by a previous report [4] showing that the full-length of HK/PB2 impairs the formation of RNP in a background of WSN/PB1, PA, NP, and vRNA. As one of the possible mechanisms for the inhibitory effect of the HK/Frag.10, we presumed that HK/Frag.10 might compete with PB2 for binding to the PB1-PA dimer and the resulting defective 3P complex might inhibit the formation of RNP, although other mechanisms should also be considered.

A primer extension assay revealed that total RNP activity, not only transcription but also replication, was inhibited by HK/Frag.10 (Figure 6). As a plaque assay showed (Figure 7), viral replication was also dose-dependently inhibited by the HK/Frag.10. These results indicate that the HK/Frag.10 inhibits total activity of the influenza virus. To assess the accuracy of the inhibitory mechanism of this fragment on RNP activity and viral replication, a highly purified peptide of HK/Frag.10 should be prepared, but this was not possible at the present because of the difficult solubility and mass expression of this peptide. A more thorough investigation into the mechanism of the inhibitory effect would require the building of a highly expressed purification system for this peptide.

Our results indicate that the N-terminal fragment of the HK (H5N1) PB2 subunit could become an effective inhibitor for the replication of influenza virus. Moreover we can suggest that both the PB1 and NP binding domains in the fragment may be necessary for a broad-spectrum inhibition. Much research about influenza virus must be conducted because influenza virus can easily develop into new pandemic strains that may have a resistance to the current drugs.
